# Nuclear translocation of beta catenin in patients with Rathke cleft cysts—clinical and imaging characteristics and risk of recurrence

**DOI:** 10.1007/s00701-023-05733-0

**Published:** 2023-08-02

**Authors:** Michael Schmutzer, Jun Thorsteinsdottir, Jonathan Weller, Walter Rachinger, Christian Schichor, Niklas Thon, Moritz Ueberschaer

**Affiliations:** Department of Neurosurgery, LMU University Hospital, LMU Munich, Marchioninistr 15, 81377 Munich, Germany

**Keywords:** Rathke cleft cysts, Transsphenoidal approach, Beta catenin, Endocrinological, Functional outcome

## Abstract

**Purpose:**

Although Rathke cleft cysts (RCC) are benign lesions of the sellar region, recurrence is frequent after surgical treatment. Nuclear translocation of ß-catenin (NTßC), a key effector of the wnt-signaling pathway that is responsible for cell renewal, has been shown to act as a proto-oncogene and is considered to be a potential risk factor for increased recurrence in RCC. In this study, we analyzed a surgically treated cohort into patients with and without NTßC expression in order to identify clinical and imaging differences and further evaluate the risk of recurrence.

**Methods:**

Patients with resection of RCC between 04/2001 and 11/2020 were included. Histological specimens were immunohistochemically stained for ß-catenin. Study endpoints were time to cyst recurrence (TTR) and functional outcome. Functional outcome included ophthalmological and endocrinological data. Furthermore, MRI data were assessed.

**Results:**

Seventy-three patients (median age 42.3 years) with RCC underwent mainly transsphenoidal cyst resection (95.9%), 4.1% via transcranial approach. Immunohistochemical staining for ß-catenin was feasible in 61/73 (83.6%) patients, with nuclear translocation detected in 13/61 cases (21.3%). Patients with and without NTßC were equally likely to present with endocrine dysfunction before surgery (*p* = 0.49). Postoperative new hypopituitarism occurred in 14/73 (19.2%) patients. Preoperative visual impairment was equal in both groups (*p* = 0.52). Vision improved in 8/21 (33.3%) patients and visual field deficits in 22/34 (64.7%) after surgery. There was no difference in visual and perimetric outcome between patients with and without NTßC (*p* = 0.45 and *p* = 0.23, respectively). On preoperative MRI, cyst volume (9.9 vs. 8.2 cm^3^; *p* = 0.4) and evidence of hemorrhage (30.8% vs. 35.4%; *p* = 0.99) were equal and postoperative cyst volume decreased significantly in both groups (0.7 vs. 0.5 cm^3^; *p* < 0.0001 each). Cyst progression occurred in 13/73 (17.8%) patients after 39.3 ± 60.3 months. Cyst drainage with partial removal of the cyst wall resulted in improved recurrence-free survival without increasing the risk of complications compared with cyst fenestration alone. Patients with postoperative diabetes insipidus had an increased risk for recurrence according to multivariate analysis (*p* = 0.005). NTßC was evident in 4/15 patients (26.7%) and was not associated with a higher risk for recurrence (*p* = 0.67).

**Conclusion:**

Transnasal transsphenoidal cyst drainage with partial removal of the cyst wall reduces the risk of recurrence without increasing the risk of complications compared with fenestration of the cyst alone. Patients with postoperative diabetes insipidus seem to have an increased risk for recurrence. In contrast, NTßC was not associated with a higher risk of recurrence and did not provide stratification for clinically distinct patients.

**Supplementary Information:**

The online version contains supplementary material available at 10.1007/s00701-023-05733-0.

## Introduction

Rathke cleft cysts (RCC), also known as cysts of the pars intermedia, account for 6–10% of all symptomatic sellar lesions. They arise from remnants of the embryonic Rathke’s pouch between the adeno- and neuropituitary and are benign lesions of the sellar region [13]. Patients usually present with headaches, visual disturbances, or endocrine dysfunction [1, 32]. While asymptomatic RCC are controlled by cranial contrast-enhanced magnetic resonance imaging (MRI), symptomatic lesions are surgically treated mostly via endosocopic or microscopic transsphenoidal approach [2, 8, 10, 25]. Postoperative improvement of symptoms has been reported in over 80% of cases [3, 19]. However, the recurrence rate is high at 18–39.6% [1, 20, 29].

With the goal of establishing optimal treatment and follow-up strategy, several attempts have been made to identify risk factors for recurrence of RCC.

An aggressive surgical approach with resection of the cyst wall might be associated with a lower recurrence rate [24]. However, the more aggressive the surgical approach, the higher the risk of postoperative hypopituitarism including diabetes insipidus and CSF leaks [39]. Therefore, some authors consider decompression and biopsy as the optimal surgical strategy in RCC [3, 39]. Preoperative cyst size, with larger cysts having a higher recurrence rate, also appears to be a risk factor for recurrence [33]. In addition, several studies have shown that histologic evidence of squamous metaplasia in the cyst wall is associated with an increased risk of recurrence [20, 33]. In single cases of recurrent cysts, immunohistochemical nuclear translocation of the cell-cell adhesion protein β-catenin (NTßC) was detected [11, 16, 30]. ß-Catenin is the key nuclear effector of the wnt signaling pathway that is responsible for cell renewal and regeneration. An imbalance in the structural and signaling properties of β-catenin is associated with an increased incidence of cancer and tumor progression [35]. Cysts with NTßC are also considered transitional forms to adamantinomatous craniopharyngiomas due to their common ectodermal root [6, 12, 14, 18, 22, 26].

The purpose of this study is to investigate whether patients with RCC and immunohistochemical NTßC have different clinical and imaging characteristics compared with patients without NTßC and whether these patients are at increased risk for recurrence.

## Methods

### Patient population

After approval by the local ethics committee of the Ludwig Maximilians University in Munich (reference number 21-0271 (03/30/2021)), the tumor registry of the Department of Neurosurgery was screened for all consecutively treated patients with RCC between 2001 and 2021. MR imaging and clinical data, including laboratory results of pituitary hormone function and ophthalmologic examination results, were frequently available at routine intervals (preoperatively and 7 days, 6 weeks, 12 months, and later after surgery). For functional outcome analysis, outcome parameters were compared with preoperative findings. Tissue of the surgical treated cases saved in the neuropathological database was reevaluated with immunohistochemical staining. All patients respectively their parents gave informed consent.

### Magnetic resonance imaging (MRI) protocol

Standard MRI routinely included axial T2-weighted sequence (with slice thickness of 2 mm), 3-dimensional T1-weighted sequences before and after intravenous administration of gadopentetate dimeglumine (Magnevist; Schering Corporation, Kenilworth, NJ) (0.1-mmol/kg body weight), and constructive interference in steady-state (CISS) sequences (with slice thickness of 1 mm each) with axial, sagittal, and coronal reconstructions each, normally performed on 1.5- or 3.0-T scanners (Magnetom Symphony, Siemens, Erlangen; Signa HDxt; GE Healthcare, Little Chalfont, UK). Cyst size was calculated by volumetry. Semi-manual segmentation of pre- and postoperative T2/CISS images was performed using the SmartBrush tool of the Elements Brainlab software (Brainlab, Munich, Germany).

An increase in postoperative cyst volume of more than 25% or symptomatic progression of the residual cyst was defined as recurrence. If postoperative imaging did not indicate a residual cyst, any new sellar cystic pathology during follow-up was considered a cyst recurrence.

### Ophthalmological data

Ophthalmological examinations and tests included visual acuity measurements and perimetry of the visual field preoperatively/at initial diagnosis (ID) and at routine time points (6 weeks, 12 months, 24 months, and later/last follow-up (FU)) after treatment evaluated by an ophthalmologist blinded to the treatment. Deficits in visual acuity were classified as mild (0.9–0.5) or severe (0.4–0), and visual field deficits as partial anopsia or complete hemianopsia. A postoperative increase in visual acuity of >0.2 and/or a decreased visual field defect was defined as an improvement, whereas a postoperative decrease in visual acuity of >0.2 and/or an increased visual field defect was assumed as deterioration. Otherwise, visual performance was classified as unchanged.

### Endocrinological data and metabolic outcome

Preoperatively and at 6 weeks, 12 months, 24 months, and later after surgery, basal serum levels of growth hormone, insulin-like growth factor 1, adrenocorticotropic hormone, baseline cortisol, prolactin, luteinizing hormone, follicle-stimulating hormone, testosterone and estradiol, thyrotropin, and thyroid hormones (fT3 and fT4) were measured. Neurohypophyseal function was measured by serum sodium and osmolality levels, fluid intake and output, and urine specific gravity level. Endocrinological deficits were pre- and postoperatively classified as anterior or posterior hypopituitarism, which includes complete and incomplete insufficiencies, accordingly to the impacted lobe. In case of partial or complete involvement of both lobes, insufficiency was defined as panhypopituitarism. Hormonal insufficiency according to each axis was considered to require substitution; otherwise, the hormonal axis was not considered to be insufficient. Postoperative improvement was assumed in case of complete recovery of at least 1 affected hormonal axis and at least unchanged status of the remaining axes. A new complete/partial insufficiency of each hormonal axis postoperatively was classified as deterioration. Otherwise, the endocrinological function was classified as unchanged.

### Treatment protocol

Treatment decisions were invariably made by the interdisciplinary tumor board. Symptomatic cysts were principally approached via the transnasal transsphenoidal route. In individual cases, a transcranial approach was chosen based on the patient’s anatomical conditions or the cyst configuration. Cysts were either drained by excision of a small window of the cyst wall (simple fenestration), or patients underwent augmented cyst wall resection. In these cases, the cyst wall was removed except for the parts adherent to the diaphragm and/or pituitary gland to avoid CSF leak or new hormone deficiency. Samples of the cyst wall were obtained from all patients. Closure was performed without fascial or fat grafts to reduce the risk of cyst recurrence [13]. A simple onlay technique with synthetic material was used, as previously described by our group for pituitary adenomas [34]. Watchful waiting of RCC was considered, if patients were asymptomatic and/or the cyst showed no compression of the optic chiasm.

### Risk assessment

Perioperative morbidity rates were determined according to all documented medical, neurological, and approach-related adverse events. Transient and permanent deficits were differentiated. Functional morbidity was analyzed separately. Courses of disease were distinguished as complicated or unremarkable.

### Immunohistochemical staining

The surgical specimens were immediately fixed in 4% buffered formalin and embedded in paraffin. Sections 3–5-μm thick were stained with hematoxylin and eosin (H&E). In addition, PAS reactions according to standard protocols as well as immunohistochemical (IHC) stainings against CK7 (Ks 7.18; Progen, Heidelberg, Germany), CK20 (Ks 20.8; Progen, Heidelberg, Germany), and ß-catenin (Clone 14; RTU, Roche Diagnostics Corporation, Indianapolis, USA) were performed. An additional dilution of the ß-catenin antibody was not performed. Standardized staining protocols were used to ensure sufficient specificity. Furthermore, in the first step, positive and negative controls were defined as references by an experienced neuropathologist. In the second step, the IHC stains were evaluated and compared with the references. In this way, positive and negative stains could always be evaluated appropriately.

### Statistical methods

The reference point of this study was the date of the first surgery. Last follow-up (FU) date was November 2020. Primary endpoints were date of cyst progression, functional outcome, and complications related to surgery. For survival analyses, Kaplan-Meier survival estimates were generated, and log-rank tests were calculated to describe cyst recurrence. Overall and particular recurrence progression rates were specified by number of patients/total case number, respectively. Results were tested by using a 2-way analysis of variance (ANOVA), Student’s *t*-test, and Fisher’s exact test. For risk factor analyses, uni- and multivariate tests were conducted. For correlation analyses, Pearson’s coefficient *r* was determined. GraphPad PRISM8.0d software was used for statistical analysis (GraphPad, San Diego, CA, USA). Statistical significance was set at *p* < 0.05.

## Results

### Patient characteristics and study population

**Fig. 1:**
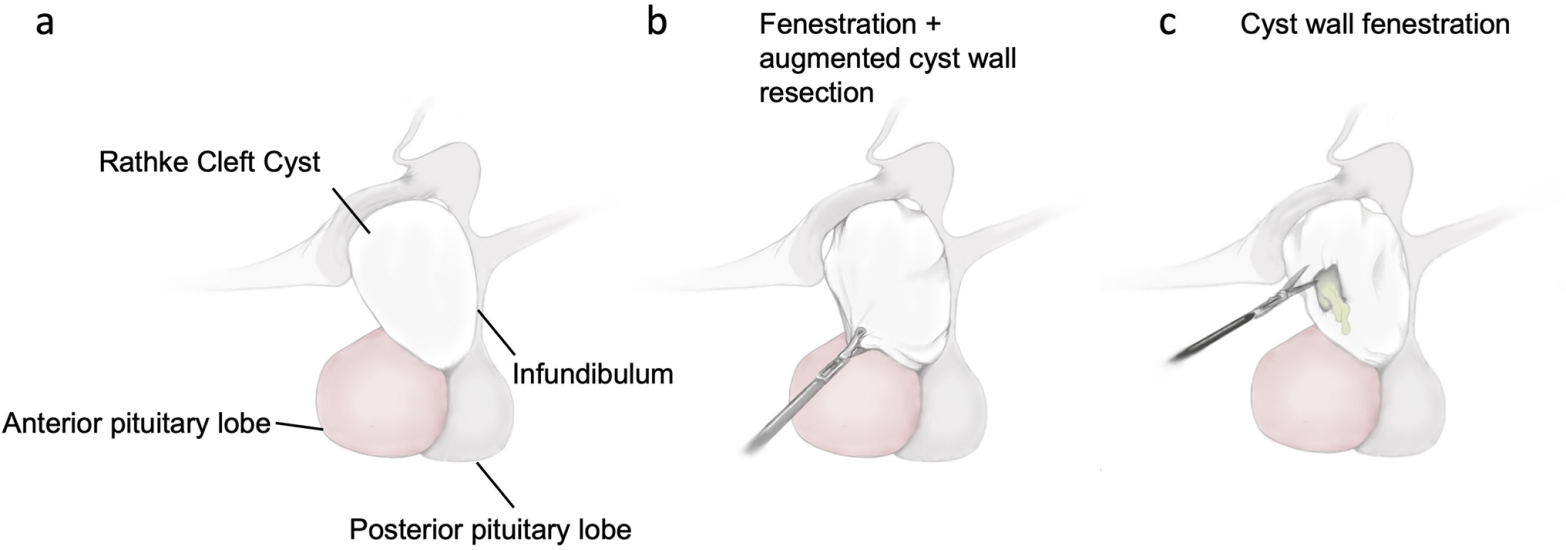
Schematic illustration of the anatomical site of the sellar region (**a**) and surgical approaches using fenestration with augmented cyst wall resection (**b**) and cyst wall fenestration alone (**c**)

One hundred forty patients with RCC were identified with 73 patients (25 male, 48 female) undergoing surgery. All 73 patients were primarily included at the time of their first surgery. Median age at first surgery was 42.3 years (range 6.8–84.2 years). Mean FU for surgically treated patients was 50.0 ± 55.7 months. In 39 cases, the cysts were suprasellar, and in 34 cases intrasellar. Initial surgery was performed via the transnasal transsphenoidal approach in 70 cases (95.9%) and via the transcranial approach in 3 patients (4.1%). In total, 65 (89.0%) fenestrations with an augmented cyst wall resection (Fig. 1b) and 8 (11.0%) simple cyst wall fenestrations were conducted (Fig. 1c).

**Fig. 2:**
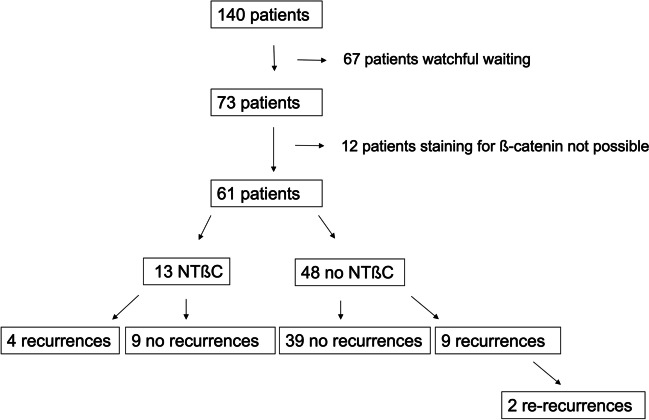
Consort diagram showing patient selection and rates of recurrence depending on NTßC status

**Fig. 3:**
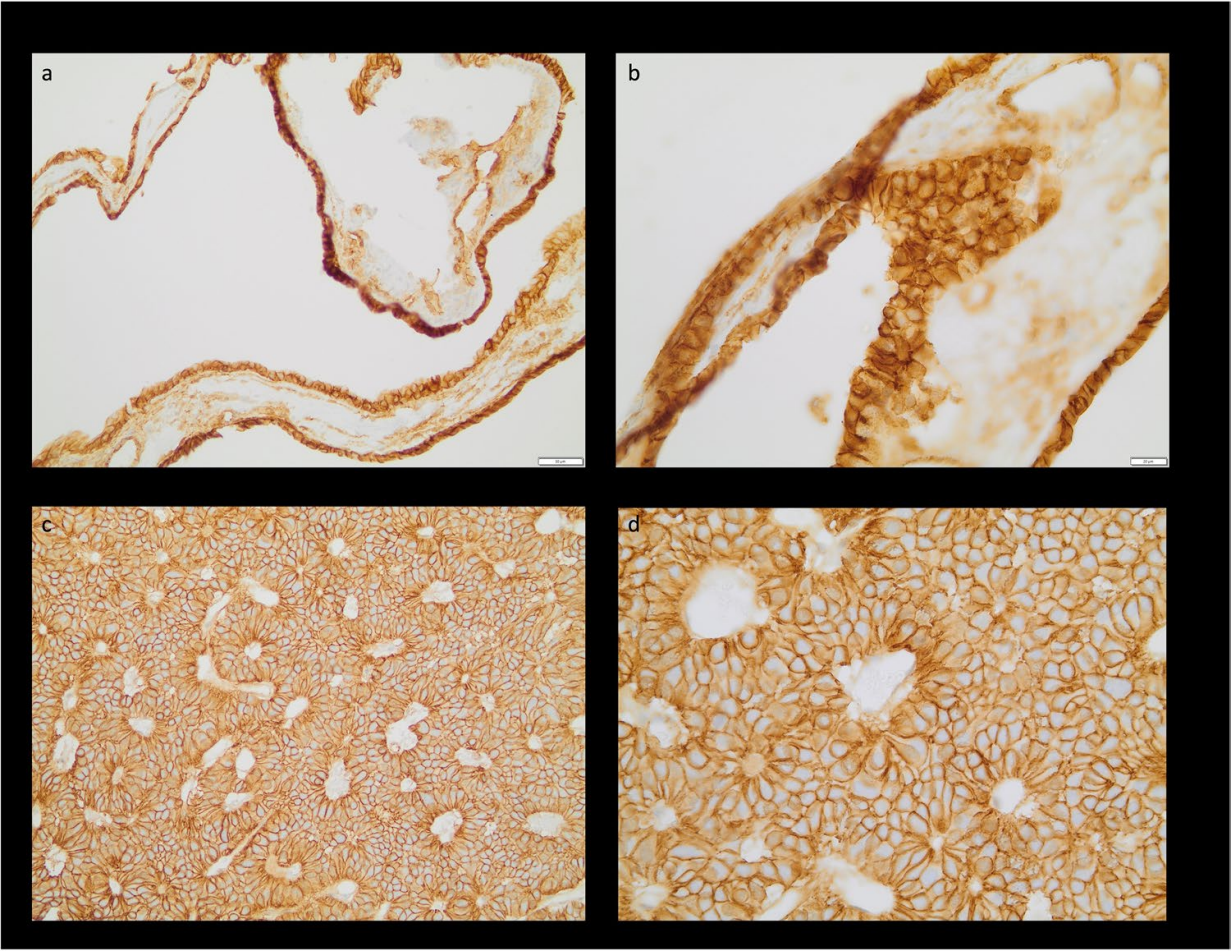
Specimen of a RCC with positive immunhistochemical staining for NTßC in ×20 magnification (**a**) and ×40 magnification (**b**). Representative negative IHC with only membranous positive ß-catenin staining at ×20 (**c**) and ×40 (**d**) magnification

Out of 73 resected RCC, 61 IHC data for NTßC were available for further evaluation (Fig. 2). CK7 and 20 were stained in 100% of cases positively. We found positive IHC for NTßC (Fig. 3) in 13/61 cases (21.3%). Successful immunostaining was independent of simple fenestration of the cyst wall or extended resection of the cyst wall (*p* = 0.99). A NTßC occurred less frequently in male patients (30.8%). Demographic data are shown in Table 1.

**Table 1:** Comparison of epidemiological parameters and MR imaging characteristics of patients with RCC with and without NTßC

Parameters	NTßC	No NTßC	*p*-value
Total, *n* (%)	13 (21.3)	48 (78.7)	**<0.0001**
Sex, *n* (%)			
Female	9/13 (69.2)	32/48 (66.7)	0.99
Male	4/13 (30.8)	16/48 (33.3)	
Serum Na^+^ (mmol/l)			
Mean **±** SD	140.3 ± 1.3	139.3 ± 4.6	0.5
Prolactin (μU/ml)			
Mean **±** SD	437.0 ± 266.5	653.2 ± 1055	0.5
Age (yrs)			
Mean **±** SD	41.9 ± 24.7	45.6 ± 19.4	0.6
FU (mo)			
Mean **±** SD	30.1 ± 31.8	49.5 ± 58.7	0.3
PFS (mo)			
Mean **±** SD	25.9 ± 30.5	38.0 ± 49.9	0.4
Cyst localization			
Intrasellar	3/13 (23.1)	25/48 (52.1)	0.1^a^
Suprasellar	10/13 (76.9)	23/48 (47.9)	
Contrast enhancement	13/13 (100)	48/48 (100)	0.99
Hemorrhagic cyst	4/13 (30.8)	17/48 (35.4)	0.99
T1 intensity			
Hyperintensity	7/13 (53.8)	18/48 (37.5)	0.3^a^
Hypointensity	6/13 (46.2)	30/48 (62.5)	
T2 intensity			
Hyperintensity	11/13 (84.6)	45/48 (93.8)	0.3^a^
Hypointensity	2/13 (15.4)	3/48 (6.3)	
Repeat surgery	3/13 (23.1)	10/48 (20.8)	0.99

^a^Fisher’s exact test

### Clinical symptoms and functional outcome

Preoperatively, 14/73 patients (19.2%) had anterior hypopituitarism, 2/73 patients (2.7%) had posterior hypopituitarism, and 1/73 patient (1.4%) suffered from panhypopituitarism. New hormonal insufficiency developed in 14/73 patients (*p* = 0.02) after surgery. Overall, the corticotropic axis was most frequently affected in 24 out of 31 patients with pituitary dysfunction (*p* = 0.0008) (Table 2). The incidence of new hypopituitarism was similar after fenestration of the cyst compared with partial resection of the cyst wall. Frequency of hypopituitarism did not differ between patients with (38.5%) versus without (25.0%) positive β-catenin expression (*p* = 0.49). Postoperatively, pituitary dysfunction was equally common in RCC with and without NTßC (suppl. table 1).

**Table 2:** Comparison of clinical symptoms, endocrinological, visual, and perimetrical findings before and after surgery

	Pre-OP *n* (%)	Post-OP *n* (%)	*p*-value
Symptoms			
Headache	37 (26.4%)	6 (4.3%)	**<0.0001**
Trigeminal neuralgia	1 (0.7%)	1 (0.7%)	0.99
Diplopia	16 (11.4%)	3 (2.1%)	**0.0024**
Endocrinological outcome			
No deficiency	56 (40.0%)	42 (30.0%)	**0.02**
Anterior hypopituitarism	14 (10.0%)	23 (16.4%)	0.13
Posterior hypopituitarism	2 (1.4%)	3 (2.1%)	0.99
Panhypopituitarism	1 (0.7%)	5 (3.6%)	0.21
Visual outcome			
Mild deficit	18 (12.9%)	12 (8.6%)	0.31
Severe deficit	3 (2.1%)	2 (1.4%)	0.99
No deficit	52 (37.1%)	59 (42.1%)	0.24
Perimetrical outcome			
Partial anopsia	16 (11.4%)	8 (5.7%)	0.11
Hemianopsia	18 (12.9%)	4 (2.9%)	**0.002**
No deficit	39 (27.9%)	61 (43.6%)	**0.0002**

Preoperatively, 18 patients (24.7%) had mild and 3 (4.1%) had severe visual loss. Visual impairments were equally frequent in both groups (*p* = 0.5). Postoperatively, vision improved in one (7.7%) of ß-catenin positive patients compared with 5 (10.4%) of ß-catenin negative patients (*p* = 0.7).

Thirty-four out of 73 patients (46.6%) suffered from visual field disturbances before surgery. Microsurgical therapy resulted in a significant reduction in the number of patients with hemianopsia (*p* = 0.002) as well as an increase in the number of patients without deficit (*p* = 0.0002) (Table 2). Preoperative visual field disturbances were evident in 7/13 patients with NTßC (*p* = 0.99). Postoperatively, perimetry improved in 3/7 (42.9%) of ß-catenin positive patients compared with 17/27 (63.0%) of ß-catenin negative patients (*p* = 0.99) (suppl. table 2).

### Imaging characteristics and volumetric data

All cysts showed circumferential contrast enhancement on initial cMRI. Spontaneous cyst hemorrhages were seen in 21/73 (28.8%) patients before surgery. In the absence of hemorrhage, the majority of cysts were hypointense in T1 sequences (86.5%) and hyperintense in T2 sequences (94.2%). The occurrence of cyst hemorrhages (30.7% vs. 35.4%; *p* = 0.99) and the preoperative cyst volume (9.9 ± 8.0 cm^3^ vs. 8.2 ± 6.2 cm^3^, *p* = 0.4) were equal in patients with and without NTßC.

**Fig. 4:**
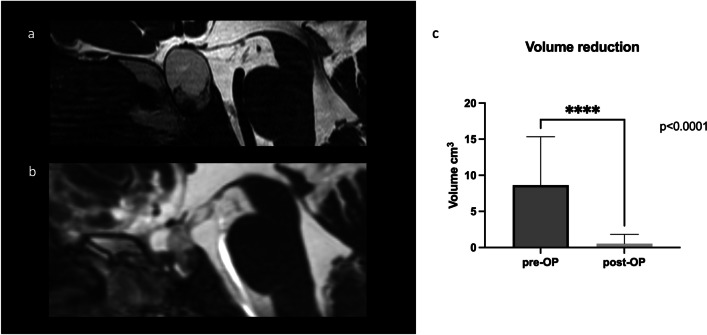
Exemplary sagittal T2 MRI before (**a**) and after (**b**) transnasal transsphenoidal decompression of RCC (**c**) shows a significant reduction in cyst volume by surgery in the entire patient cohort

Cyst volume significantly decreased after surgery in both groups (0.7 ± 1.3 cm^3^ vs. 0.5 ± 1.2 cm^3^; *p* < 0.0001 each) (Fig. 4a–c).

### Perioperative morbidity

In 99 surgeries, including 26 second interventions for cyst recurrence and revision surgeries for complications, 8/99 (8.1%) CSF leaks occurred always after transsphenoidal microsurgical resection. Six out of 8 patients underwent a total of 9 transsphenoidal microscopical revision surgeries and 2/8 patients were treated with lumbar CSF drainage. Furthermore, two postoperative intrasellar abscesses (2.0%) after transsphenoidal microsurgery occurred, both of which were surgically revised and subsequently treated with intravenous antibiotics. In 12 (12.1%) cases, transient postoperative electrolyte disorders were diagnosed. Perioperative complications did not differ significantly among patients treated by solely fenestration and with augmented cyst wall resection. Patients with NTßC did not have more surgical complications (*p* = 0.2).

### Outcome

RCC recurrence occurred in 13 of 73 (17.8%) cysts after a mean time of 52.4 ± 72.1 months after initial surgery (median 17 months, range 3.6–187.2 months). In case of recurrence, reoperation was performed in all 13 patients. In 2 of these patients, repeat transsphenoidal surgery was required because of cyst recurrence after a mean period of 26.1 months. NTßC had no effect on the need for repeat surgery (3/13 (23.1%) vs. 10/48 (20.8%); *p* = 0.99). One patient received adjuvant stereotactic radiosurgery. Symptoms at revision surgery included headache in 9/15 (60%), hemi- or partial anopsia in 3/15 cases (20.0%), fatigue in 2/15 (13.3%), and abducens palsy or trigeminal neuralgia in 1 case each (6.7%). We found NTßC in 4 of 15 recurrent RCCs (26.7%).

**Fig. 5:**
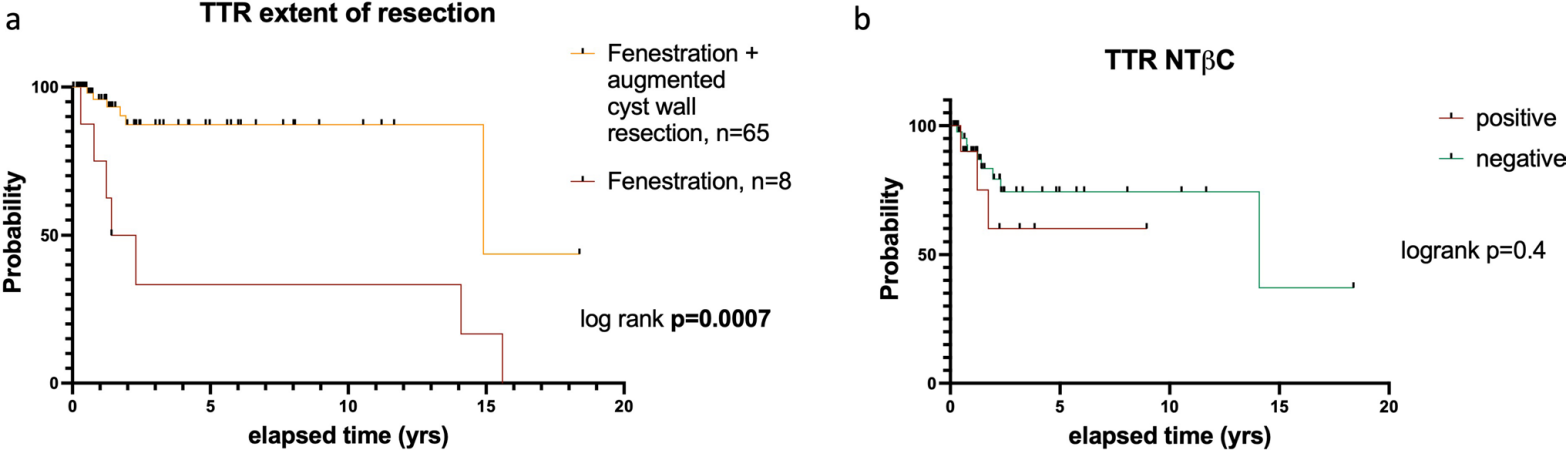
PFS of patients with RCC depending on extent of resection (**a**) and according to NTßC status (**b**)

TTR after cyst fenestration and augmented cyst wall resection was higher than after cyst fenestration alone (55.7 ± 76.0 months vs. 41.0 ± 46.8 months; *p* = 0.0007, HR=0.23, Fig. 5a). The 1-, 2-, 5-, and 10-year probabilities without cyst recurrence were 94.0%, 85.6%, 85.6%, and 85.6% after cyst fenestration with cyst wall resection compared with 75.0%, 50.0%, 33.3%, and 33.3% after simple fenestration of the cyst. In contrast, a comparison of TTR between NTßC positive and negative RCC showed no significant difference (*p* = 0.4) with PFS of 25.9 ± 30.5 months and 38.0 ± 49.9 months (Fig. 5b), respectively.

### Risk factors for recurrence

Univariate analysis revealed that impaired preoperative visual acuity (*p* = 0.026), perimetric deficits (*p* = 0.0001), postoperative diabetes insipidus (*p* = 0.003), hypopituitarism (*p* = 0.024), and repeat surgery (*p* < 0.0001) were risk factors for recurrent and clinically progressive RCC. Only postoperative diabetes insipidus (*p* = 0.005) and repeat surgery (*p* < 0.0001) remained as significant risk factors for recurrent RCC in multivariate analysis. NTßC was not associated with a higher risk of recurrence (*p* = 0.67) (Table 3).

**Table 3:** Uni- and multivariate analyses for risk factors for recurrent RCC

Characteristic	Univariate 95% CI	*p*-value	Multivariate 95% CI	*p*-value
RCC recurrence				
Visual acuity pre-OP	0.024 to 0.34	**0.026**	−1.254 to 0.5382	0.42
Perimetrical deficits pre-OP	0.1633 to 0.4648	**0.0001**	−0.2526 to 0.7016	0.35
Volume pre-OP	0.1595 to 0.2289	0.72		
Volume reduction	−0.386 to 0.158	0.39		
Cyst localization	−0.05638 to 0.2809	0.19		
Hemorrhage	−0.04972 to 0.2871	0.16		
Diabetes insipidus post-OP	0.092 to 0.406	**0.003**	19.51 to 100.2	**0.005**
Hormone deficiency post-OP	0.027 to 0.353	**0.024**	−45.3 to 1.44	0.06
Age	0.134 to 0.204	0.68		
Sex (m vs. f)	−0.084 to 0.253	0.32		
NTßC	−0.2049 to 0.3101	0.67		
Repeat surgery	0.05680 to 0.2826	**<0.0001**	0.4315 to 0.7683	**<0.0001**

## Discussion

Despite being benign lesions, recurrence occurs in up to 39.6% of surgically treated RCC [20]. Aggressive surgery with resection of the entire cyst wall may reduce the risk of recurrence but increases the risk of complications [24, 39]. Therefore, identification of risk factors for recurrence is critical for both surgical strategy and tailored follow-up management.

In 2013, Ogawa et al. [30] reported on a cohort of 12 patients with RCC. There were 5 patients with cyst recurrence that all showed NTßC expression in IHC. In this study, we aimed to investigate firstly in a huger patient cohort if patients with NTßC have an increased risk for recurrence and if these patients have a different clinical presentation and postoperative course.

Age and sex distribution with a male to female ratio of 1.4:1 in our cohort was characteristic for RCC [2, 28]. Headaches and endocrine dysfunction were typical symptoms at first diagnosis and frequency was comparable with previous publications [17, 21]. In line with the findings of Eymann et al. [9], MRI showed a mixed intensity of RCC in T1 and T2. The surgical approach was mainly transsphenoidal (95.9%) according to the standard of care. The recurrence rate of 17.8% was relatively low but within the range of previously reported recurrence rates [5, 7, 37]. Ogawa et al. and Hofmann et al. [16, 30], however, discussed an increased risk of recurrence in cystic lesions of the sellar region with NTßC in a cohort of 12 and 30 patients with RCC. The patient cohort of our study was much larger with 61 patients. However, extension beyond the follow-up period of 39.3 months might result in a higher rate of recurrences in here, too.

Suprasellar located cysts were associated with hemorrhages and T1 hyperintensity on MRI. Despite these specific imaging findings, clinical presentation before surgery as well as the postoperative course of patients with and without NTßC did not differ significantly.

Our results showed recurrence of cysts in 26.7% of RCC with and in 18% without NTßC. However, statistically, there was no increased risk for recurrence in patients with NTßC (*p* = 0.67). Therefore, our findings do not corroborate the assumption that patients with NTßC have an increased risk for recurrence. Although 69.2% of all patients with NTßC were female, this could not be confirmed as a significant risk factor for RCC recurrence in univariate analysis.

While there is evidence to suggest that sex hormones can influence the Wnt/ß-catenin signaling pathway [4, 36], the relationship between sex and NTßC is currently unclear. The complex interactions between sex hormones and beta-catenin signaling likely depend on the specific cellular and molecular context [23].

The trend toward an inverse relationship between sex and NTßC in our retrospective patient cohort must be interpreted cautiously in light of the complex mechanisms of the Wnt/ß-catenin pathway and the relatively small number of patients. Larger studies need to be performed to determine whether this observation is reproducible and potentially of clinical significance. In addition, further research is needed to understand the underlying mechanisms.

Previously, risk factors for recurrence like cyst remnants on postoperative MRI or squamous metaplasia of the cyst wall have been described [20, 27]. In our patient cohort, risk factors for recurrence were preoperative visual symptoms and postoperative hormonal deficiencies according to univariate analysis. Postoperative diabetes insipidus was the only parameter that significantly increased the risk for recurrence according to multivariate analysis. It might be possible that these cysts are more infiltrative variants that either require increased manipulation during resection or are otherwise biologically distinct. However, our data do not provide evidence for this hypothesis.

The impact of the surgical procedure on the risk of recurrence has been studied extensively. Although Mendelson et al. described advantages of the endoscopic technique over the microscopic approach [28], we do not consider it a drawback of the study that the patients underwent microsurgery, as there is a wide range of recurrence rates in the literature and our recurrence rates are in the low to intermediate range. This is supported by the results of Kinoshita et al., who did not find a higher risk for recurrence depending on the surgical technique [20].

In our cohort, a comparison of cyst fenestration alone with partial resection of the cyst wall was found to be associated with longer recurrence-free survival (55.7 ± 76.0 months vs. 41.0 ± 46.8 months) without increasing the risk of postoperative complications. Accordingly, our results support the surgical strategy of partially removing the cyst wall and draining the cyst contents.

In line with previous publications, we could show that surgery is very effective in improving headache and visual symptoms [7, 15, 32, 38]. However, new hormone deficits especially of the corticotropic (*p* < 0.008) and thyreotropic (*p* = 0.057) axes occurred in a relevant number of cases. Since other studies [31, 37] report comparable complication rates, we recommend a cautious indication for surgery.

Previous studies reported that NTßC in cases of adamantinomatous cystic craniopharyngeomas was associated with worse outcome and a higher rate of recurrence, and suggested NTßC as a distinct differentiation marker for craniopharyngeomas [12, 16, 37]. In total, 21.3% of RCC with NTßC argues against it being a unique marker for distinguishing the two entities, as Hofmann et al. [16] discuss a transitional form between RCC and craniopharyngioma may be possible. However, within our subset of RCC patients with NTßC, no other clinical or imaging features emerged that could stratify for a specific subset of cysts.

## Conclusion

Transnasal transsphenoidal cyst drainage with partial removal of the cyst wall reduces the risk of recurrence without increasing the risk of complications compared with fenestration of the cyst alone. Patients with postoperative diabetes insipidus seem to have an increased risk for recurrence. In contrast, NTßC was not associated with a higher risk of recurrence and did not provide stratification for clinically distinct patients.

## Supplementary information

ESM 1 (DOCX 21 kb)
