# Supplementary material for: Nuclear translocation of beta catenin in patients with Rathke cleft cysts—clinical and imaging characteristics and risk of recurrence
Source: Acta Neurochir (Wien). 2023 Aug 2;165(9):2435–44. doi: 10.1007/s00701-023-05733-0 (PMC10477103; doi:10.1007/s00701-023-05733-0)
Supplement: Supplementary file 1 — (DOCX 21 kb) [file 701_2023_5733_MOESM1_ESM.docx]

Supplementary Tables:

Suppl. table 1: Pre- and postoperative endocrinological deficiencies of RCC patients according to their β-catenin status.

| **Deficiency** | **Pre-OP**  **NTßC**  **n=13** | **Pre-OP**  **No NTßC**  **n=48** | **p-value** |
| --- | --- | --- | --- |
| **Corticotropic** | 3 | 8 | 0.69 |
| **Somatotropic** | 0 | 2 | 0.99 |
| **Thyreotropic** | 2 | 7 | 0.99 |
| **Gonadotropic** | 2 | 9 | 0.99 |
| **Diabetes insipidus** | 1 | 1 | 0.38 |
| **Hyperprolactinemia** | 5 | 16 | 0.75 |
| **No deficiency** | 8 | 36 | 0.49 |
|  |  |  |  |

| **Deficiency** | **Post-OP**  **NTßC**  **n=13** | **Post-OP**  **No NTßC**  **n=48** | **p-value** |
| --- | --- | --- | --- |
| **Corticotropic** | 5 | 19 | 0.99 |
| **Somatotropic** | 1 | 4 | 0.99 |
| **Thyreotropic** | 3 | 13 | 0.99 |
| **Gonadotropic** | 1 | 10 | 0.43 |
| **Diabetes insipidus** | 1 | 5 | 0.99 |
| **Hyperprolactinemia** | 0 | 0 | 0.99 |
| **No deficiency** | 7 | 24 | 0.99 |

Suppl. table 2: Pre- and postoperative visual and perimetrical deficiencies of RCC patients according to their β-catenin status.

| **Deficiency** | **Pre-OP**  **NTßC**  **n=13** | **Pre-OP**  **No NTßC**  **n=48** | **p-value** |
| --- | --- | --- | --- |
| **Visual outcome**  Mild deficit | 4 | 13 | 0.99 |
| Severe deficit | 1 | 1 | 0.38 |
| No deficit | 8 | 34 | 0.52 |
| **Perimetrical outcome**  Partial anopsia | 2 | 11 | 0.72 |
| Hemianopsia | 5 | 13 | 0.5 |
| No deficit | 6 | 24 | 0.99 |
|  |  |  |  |

| **Deficiency** | **Post-OP**  **NTßC**  **n=13** | **Post-OP**  **No NTßC**  **n=48** | **p-value** |
| --- | --- | --- | --- |
| **Visual outcome**  Mild deficit | 3 | 8 | 0.69 |
| Severe deficit | 1 | 1 | 0.38 |
| No deficit | 9 | 39 | 0.45 |
| **Perimetrical outcome**  Partial anopsia | 3 | 5 | 0.35 |
| Hemianopsia | 1 | 2 | 0.51 |
| No deficit | 9 | 41 | 0.23 |
